# The Light Responsive Transcriptome of the Zebrafish: Function and Regulation

**DOI:** 10.1371/journal.pone.0017080

**Published:** 2011-02-15

**Authors:** Benjamin D. Weger, Meltem Sahinbas, Georg W. Otto, Philipp Mracek, Olivier Armant, Dirk Dolle, Kajori Lahiri, Daniela Vallone, Laurence Ettwiller, Robert Geisler, Nicholas S. Foulkes, Thomas Dickmeis

**Affiliations:** 1 Institute of Toxicology and Genetics, Karlsruhe Institute of Technology, Eggenstein-Leopoldshafen, Germany; 2 Max-Planck-Institut für Entwicklungsbiologie, Tübingen, Germany; 3 Heidelberg Institute of Zoology, University of Heidelberg, Heidelberg, Germany; University of Birmingham, United Kingdom

## Abstract

Most organisms possess circadian clocks that are able to anticipate the day/night cycle and are reset or “entrained” by the ambient light. In the zebrafish, many organs and even cultured cell lines are directly light responsive, allowing for direct entrainment of the clock by light. Here, we have characterized light induced gene transcription in the zebrafish at several organizational levels. Larvae, heart organ cultures and cell cultures were exposed to 1- or 3-hour light pulses, and changes in gene expression were compared with controls kept in the dark. We identified 117 light regulated genes, with the majority being induced and some repressed by light. Cluster analysis groups the genes into five major classes that show regulation at all levels of organization or in different subset combinations. The regulated genes cover a variety of functions, and the analysis of gene ontology categories reveals an enrichment of genes involved in circadian rhythms, stress response and DNA repair, consistent with the exposure to visible wavelengths of light priming cells for UV-induced damage repair. Promoter analysis of the induced genes shows an enrichment of various short sequence motifs, including E- and D-box enhancers that have previously been implicated in light regulation of the zebrafish *period2* gene. Heterologous reporter constructs with sequences matching these motifs reveal light regulation of D-box elements in both cells and larvae. Morpholino-mediated knock-down studies of two homologues of the D-box binding factor Tef indicate that these are differentially involved in the cell autonomous light induction in a gene-specific manner. These findings suggest that the mechanisms involved in *period2* regulation might represent a more general pathway leading to light induced gene expression.

## Introduction

The ability to perceive sunlight provides animals with many adaptive advantages. Light perception can be used for orientation in the environment, the location of prey and the escape from predators as well as for communication via visual signals. The daily changes in lighting conditions also represent an important time cue for the optimal temporal distribution of activities and physiological processes of the organism, which in turn enhances survival. In this case, environmental lighting signals cooperate with endogenous timers, the most important of which is the circadian clock.

Circadian clocks regulate daily changes in physiology in most organisms, ranging from cyanobacteria to humans [1]. These clocks consist of an oscillator mechanism that generates rhythms with a period of roughly 24 hours even in the absence of external cues. Molecularly, the oscillator is composed of proteins participating in a transcription-translation feedback loop (reviewed in [2], [3], [4]). In vertebrates for example, the transcription factors Clock and Bmal1 activate transcription of the *period* (*per*) and *cryptochrome* (*cry*) genes via so-called E-box elements in their promoters. The Period and Cryptochrome proteins accumulate in the cytoplasm, re-enter the nucleus and act there to inhibit the transcriptional activity of the Clock-Bmal1 complex. This reduces the transcription of the *cry* and *per* genes, hence less protein is made, and the repression is released, so that the cycle can start again. Additional feedback loops and posttranslational modifications are thought to confer the 24 h period of this mechanism and to render it more robust. This molecular clock mechanism is encountered in the neural circadian pacemaker of the brain, the paired suprachiasmatic nucleus (SCN) situated in the hypothalamus above the optic chiasm, which drives rhythms of behavior and other “centrally” regulated aspects of physiology. However, this clock mechanism is also present in most other tissues of the body, constituting so-called “peripheral” clocks. These clocks govern many aspects of cell and tissue physiology, acting either autonomously or in concert with systemic cues.

The circadian oscillator can be synchronized with (or “entrained” to) the environment via a number of different cues, including temperature, food, various chemical compounds and, perhaps most pervasively, light. In mammals, a subset of retinal ganglion cells which are intrinsically photosensitive (so-called ipRGCs, [5]) projects to the SCN. The opsin photopigment, melanopsin, is expressed in the ipRGCs and is sufficient for circadian light responses in the absence of functional rods and cones [6], [7], [8]. However, melanopsin knock-out mice still entrain to light-dark (LD) cycles when rods and cones are present, therefore the ipRGCs can also transmit light information received through the rods and cones [9]. Light induced changes in ipRGC activity are signalled via glutamate and PACAP containing projections to the neurons of the SCN, leading to acute changes in neuronal activity and also to gene expression changes mediated by the transcription factor CREB [10]. Currently poorly defined signals, which might include hormones such as glucocorticoids, as well as body temperature changes and the activity of the autonomic nervous system, then transmit timing information from the SCN to the peripheral tissue clocks [11], [12]. However, under some conditions, e.g. unnaturally timed feeding schedules [13], peripheral clocks can be “decoupled” from the SCN clock and run even in antiphase to it, revealing the principal autonomy of peripheral tissue clocks from the central pacemaker. Nevertheless, to perceive changes in environmental lighting conditions, mammalian peripheral clocks rely upon systemic signals from the SCN.

This is not the case in many other organisms. In *Drosophila*, peripheral tissue clocks respond directly to light [14]. Cryptochrome, which functions as part of the core clock feedback loop in mammals, serves as a photopigment in the fruit fly [15]. Strikingly, direct light responsiveness of peripheral clocks is also encountered in a vertebrate, the zebrafish. Clock gene expression in zebrafish organ cultures can be entrained to LD cycles [16], and even single zebrafish cell culture cells are able to entrain their clocks in response to light pulses [17], [18]. In the absence of light, rhythms in single cells continue, but drift out of phase with respect to the other cells, leading to an apparent dampening of the rhythm of the entire culture. The nature of the photoreceptor mediating this peripheral light responsiveness is still elusive, although various candidates have been proposed, including Teleost Multiple Tissue (TMT-) Opsin and Cryptochromes [19], [20]. A more recent study has implicated specifically the *cry1a* gene in mediating the effects of light on the clock. However, Cry1a is hypothesized to act as an element of the signalling pathway, rather than serving as a light receptor itself [21]. The expression of several clock genes acutely responds to light in zebrafish cells, e.g. *per1*, *per2* and *cry1a* [17], [22], [23], [24]. Light induced expression of the *per2* gene has very recently been shown to depend on D- and E-box enhancer elements within its promoter [25]. Strikingly, evidence points to the signalling pathways mediating light induced clock gene expression in fish being conserved in mammals, despite the lack of peripheral photoreception. Fibroblasts transfected with melanopsin become light sensitive and can entrain their clocks in response to exposure to LD cycles [26], [27]. Furthermore, the light responsive D- and E-box enhancer elements of the *per2* gene are conserved in chicken and mammals, including humans, and a human promoter fragment containing these elements mediates light induction of a reporter gene when transfected into zebrafish cells. One way to interpret these findings would be that, in mammals, SCN-controlled signals for the entrainment of peripheral clocks might have co-opted pathways that formerly acted downstream of the now lost peripheral light receptor [25].

Interestingly, the direct light reception in peripheral tissues might affect physiology not only indirectly, via regulating the clock, but also more directly. For example, one gene that has been shown to be light inducible already in the early embryo, even before the clock is running properly, is the DNA repair enzyme *6,4-photolyase/cry5* [28]. This regulation seems likely to have physiological relevance, since mortality caused by UV treatment was reduced when zebrafish embryos had been exposed to light prior to treatment [28]. Similar findings have recently been reported in the zebrafish z3 cell line [29].

Here, we set out to characterize light induced transcription in the zebrafish more globally by identifying light responsive genes at three levels of organization: whole larvae, heart organ cultures and cell culture cells. We identified a relatively restricted set of 117 regulated genes, the majority (90) being upregulated by light. Induced genes fell into a variety of functional categories, including genes involved in circadian clock function, DNA repair, retinal light reception and metabolism, highlighting the pervasive effects of light exposure on physiology. Strikingly, examination of the promoters of the upregulated genes revealed an enrichment for E- and D-box binding sites, indicating that the role E- and D-box binding factors play in light induction of the *per2* gene might also extend to many other light responsive genes.

## Materials and Methods

### Ethics Statement

All zebrafish husbandry and experimental procedures were performed in accordance with the German animal protection standards and were approved by the Government of Baden-Württemberg, Regierungspräsidium Karlsruhe, Germany (Aktenzeichen 35-9185.64).

### Raising adult and larval zebrafish

Adult zebrafish (Tübingen strain) were raised according to standard methods [30]. Fertilized eggs were collected within 2 h of laying, and aliquots of 20 eggs were transferred into 20 ml of E3 buffer in 25-cm^2^ tissue culture flasks [31]. The flasks were incubated in a large-volume thermostat-controlled water bath equipped with an Osram L15W/41-827 light source for 5 days in constant darkness (DD) at 25°C, then illuminated with an approximate intensity of 1,200 lux for 1 or 3 h or maintained in DD before RNA extraction. The intensity of illumination was measured with a POCKET LUX Illuminance Meter (LMT, Berlin).

### *In vitro* heart culture

*In vitro* heart cultures were carried out essentially as described before [32]. Briefly, freshly dissected tissue was placed in L15 medium supplemented with 15% fetal calf serum and with gentamycin (50 µg/ml) and Pen/Strep (100 units/ml; 100 µg/ml). Organs were dissected from fish and washed 4 times with medium (10 ml/5 hearts). 5 hearts each were placed into cell culture flasks containing 5 ml of medium and submerged in the water bath in constant darkness for 4 days, then subjected to the light pulse as performed for the larvae and finally directly processed for RNA extraction.

### Cell culture

PAC2 cell culture was carried out essentially as described before [16], [17]. Cells were seeded into 75 cm^2^ flasks and submerged in the waterbath in constant darkness for a week before light pulse treatment and RNA isolation.

### RNA isolation and microarray hybridization

Total RNA was extracted from at least three biological repeat samples per experimental condition using Trizol RNA isolation reagent (GIBCO-BRL) according to the manufacturer's instructions. Synthesis and labeling of antisense RNA was performed as recommended by the array manufacturer, using kits for double-stranded cDNA synthesis (Invitrogen), for transcription and labeling of antisense RNA (Enzo Life Sciences) and for probe purification and hybridization controls (Affymetrix). Samples were hybridized to the Affymetrix Zebrafish GeneChip, representing 15,617 probes.

### Microarray analysis

Microarray hybridization data were analyzed using scripts written in the statistical programming language R [33] supported by packages provided by the Bioconductor project [34]. Background correction, normalization and probe set summarization were performed using the robust multi array algorithm with background adjustment (gcrma, [35]). Two methods were employed to detect genes differentially expressed in response to light exposure. First we used linear models and a moderated *t*-statistic from the package *limma* [36]. Multiple testing correction was performed using Benjamini and Hochberg false discovery rate (FDR) [37] and genes with an adjusted *p*-value of ≤0.05 were considered differentially expressed. Secondly, we used the meta-analysis technique, Rank Product [38], to generate a non-parametric statistic that detects genes consistently highly ranked in the comparisons between light exposed and control samples. All data is MIAME compliant, and raw and normalized data were stored in the ArrayExpress data base (http://www.ebi.ac.uk/microarray-as/ae/, accession-no. E-MTAB-381).

Annotations for the differentially expressed genes were retrieved with the Affymetrix probe set IDs using BioMart version 0.7 [39], querying the dataset of zebrafish genes based on the genome release Zv8 in the Ensembl database (release 56). In the cases where no Ensembl Gene IDs were assigned, we examined the location of the probe set by BLAST to check whether they were located 5′ or 3′ to an annotated gene. If there was a unique BLAST hit close to a gene annotation, the corresponding Ensembl Gene ID was assigned to the probe set and marked as a 5′ or 3′-hit. The Ensembl Gene IDs were subsequently used to assign the corresponding ZFIN IDs and Entrez Gene IDs. For some probes, including the two most highly downregulated ones, no annotated sequence could be identified in the Zv8 release with either strategy, and they were therefore excluded from the subsequent analyses. Where no particular references are given, the gene description is based on information contained in the NCBI (Gene, RefSeq and OMIM) and ZFIN databases.

### Cluster analysis

Cluster 3.0 [40] and Java TreeView 1.1.5r2 [41] were used for cluster analysis. Hierarchical clustering was performed using a centroid linkage algorithm with an uncentered Pearson correlation similarity metric. Some genes are hit by several probes that may fall into two different clusters, with one cluster corresponding to a subset of expression patterns of the other. When counting the numbers of genes belonging to each cluster, we counted such genes only once, in the cluster that included all experimental conditions where the gene was found to be regulated. For example, *xpc* is hit by two probes, Dr.4069.1.A1_at and Dr.19728.1.A11_at. Dr.4069.1.A1_at belongs to the “larvae and cells” cluster, whereas Dr.19728.1.A11_at belongs to the “cells” cluster. The gene is thus counted only once, in the “larvae and cells” cluster.

### Gene Ontology (GO) term analysis

For the GO term enrichment analysis we used BinGO 2.4.2 [42], a plug-in for the Cytoscape 2.7.0 platform [43]. The ontology data for zebrafish were obtained from the Gene Ontology project (www.geneontology.org). The GO terms of the light-induced genes were compared to a gene universe that was restricted to the expressed genes on the microarrays that could be linked to a ZFIN ID. To identify statistically significantly overrepresented GO categories, a hypergeometric test was performed followed by FDR correction [37]. GO terms with corrected *p*-values below 0.05 were considered as significantly enriched.

### Human Disease MeSH terms and KEGG pathway enrichment analysis

The list of human orthologues (Table S4) was derived either from the corresponding ZFIN human orthologue annotation of the zebrafish genes or from manually curated reciprocal BLAST hits in the NCBI non-redundant protein database. In order to find human diseases linked with the list of human orthologues, we used LitInspector data for retrieving MeSH terms (Medical Subject Headings) in the Diseases Category [44]. MeSH terms with an adjusted *p*-value less than 0.05 as calculated by a hypergeometric test and adjusted by a simulated Benjamini and Hochberg FDR correction of 1000 multiple tests are considered as significantly enriched in the human orthologues dataset. KEGG pathway analysis was performed with the ClueGO plugin for Cytoscape [45].

### Promoteranalysis

We identified overrepresented motifs in the promoter sequences of the light induced gene set as follows: Promoter sequences were retrieved with Gene2Promoter within the Genomatix software suite. The list of promoters from the upregulated gene set was manually curated according to transcription start site locations derived from zebrafish cDNA sequences of the NCBI RefSeq or EMBL Nucleotide Sequence databases (as well as from our own 5′-RACE data in the case of *lonrf1(2of2)*, *cry1a* and *per2*). The genomic coordinates of the retrieved promoters are shown in Table S2. We next applied the Trawler-standalone pipeline [46], [47] to define enriched motifs in the promoters of the light induced gene set against a background that consisted of the promoter sequences from the entire gene universe used for the GO enrichment analysis (see above). The location of the obtained motifs in the tested gene set promoters was visualized by the Motif Align and Search Tool (MAST) within the MEME software suite [48].

### Morpholino oligonucleotide mediated knock-down

Gene knock-down experiments were performed using morpholino-modified antisense oligonucleotides (MO; Gene Tools): Cells were electroporated with 10 µM morpholino oligonucleotides (sequences: Gene Tools standard control MO, 5′-ctcttacctcagttacaatttata-3′; tef1 MO, 5′-agtgttctgttcttacagacctgat-3′; tef2 MO, 5′-tcagctttaatcatctcctaccgtc-3′) before transferring them into 6 well plates. The tef2 MO targets the splice site between Exon 1 and Intron 1 of *tef2*, and the tef1 MO targets the boundary of exon 2 and intron 2 of *tef1* [25]; knock-down efficiency was evaluated by RT-PCR. Electroporation was performed at 0.29 kV, 960 µF, using a Gene Pulser apparatus (Bio-Rad). Transfection efficiency was determined with a lissamine-tagged fluorescent control morpholino and found to be 90%. Cells were incubated for 48 h before a 3-hour light pulse and subsequent RNA isolation.

### Quantitative RT-PCR

RNA (1 µg) was reverse-transcribed using Oligo(dT) primer or random primers (Amersham Biosciences and Invitrogen) and SuperScript III reverse transcriptase (Invitrogen). mRNA levels were determined by real-time qPCR using the DNA Engine Opticon thermocycler (Bio-Rad) or StepOne Plus (Applied Biosystems) following the manufacturer's instructions. First-strand cDNA aliquots from each sample were diluted 20× and served as templates in a PCR reaction consisting of master mix, SYBR Green I fluorescent dye (Bio-Rad), and 400 nM gene-specific primers. Copy numbers were normalized using *β-actin* controls. Primer sequences are shown in Table S5.

### Bioluminescence assays

To analyze whether the motifs identified by the Trawler pipeline were able to modulate transcription in a light dependent manner, oligonucleotides containing the concatemerized motifs separated by 10 bp spacers were subcloned into pLucMCS (Stratagene) upstream of the luciferase reporter gene as previously described ([17], see Table S6 for the oligonucleotide sequences). Pac2 cells were transiently transfected in 6 well plates with FuGENE HD (Roche) according to the manufacturer's protocol. Cells from each well were transferred after 24 h into 8 wells of 96-well fluoro-assay plates (ThermoFisher Scientific, Nuncbrand). The next day, culture medium was replaced with medium containing 0.5 mM beetle luciferin (Promega) and cells were kept in darkness for 3 days before being submitted to the light regime indicated in Figure 6. Bioluminescence was monitored on a Packard TopCount NXT scintillation counter at 25°C.

For *in vivo* luciferase monitoring in larvae, the 4x E-box and 6x D-box constructs were injected into fertilized zebrafish eggs. Larvae were placed into E3 medium containing 0.5 mM beetle luciferin and kept under constant darkness for three days, then placed into a Packard TopCount NXT scintillation counter for bioluminescence monitoring under the light regime indicated in Figure 7.

## Results and Discussion

### Experimental design and global patterns of light responsive transcription

**Figure 1:**
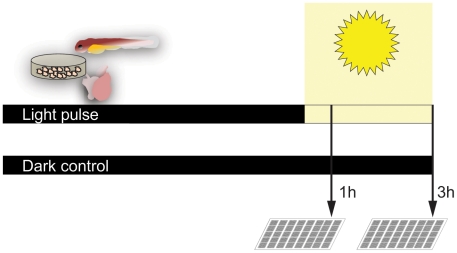
Experimental design of the study. We examined light induced gene expression at three levels of organization: The cell (PAC2 cells), the organ (cultured hearts) and the organism (larvae). Samples were kept in the dark to desynchronize their clocks before being exposed to a 1 or 3-hour light pulse or left in the dark as a control. Samples from three biological repeats per treatment and control were hybridized to Affymetrix Zebrafish GeneChips.

**Figure 2:**
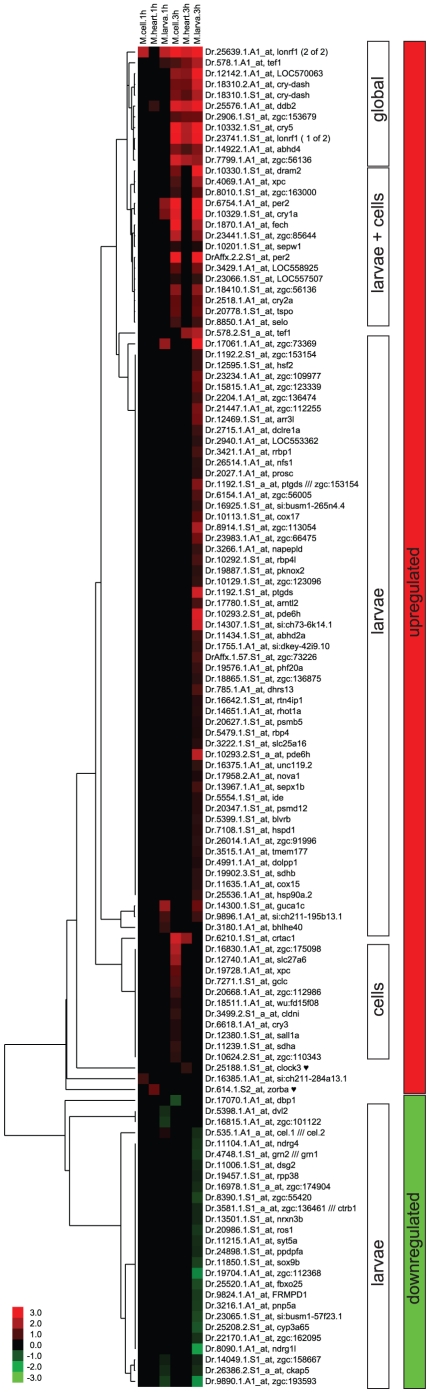
Cluster analysis of the microarray experiments. Results of cluster analysis of the microarray experiments. Upregulated transcripts are shown in red, downregulated ones in green, with the colour intensity indicating the log_2_ fold change values. Five major clusters can be identified: globally upregulated, up in larvae and cells, up in larvae only, up in cells only and downregulated in the larvae. For details see text. 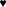, heart specific genes.

To obtain a global picture of genes regulated by light in the zebrafish, we determined genes that significantly changed their expression upon a 1- or 3-hour exposure to light in zebrafish cell cultures, heart cultures and larvae using Affymetrix microarrays (Figure 1, see Materials and Methods for details of the microarray analysis). This experimental design was expected to reveal genes immediately downstream of photoreceptors as well as a second wave of regulated genes, both at three different levels of organization: the cell, the organ and the entire animal (Figure 2 and Table S1).

Larvae were raised in constant darkness for 5 days before being subjected to a light pulse of 1 or 3 hours. Since the circadian clock is desynchronized in the larvae under these conditions [49], [50], we could examine the effect of the light pulses regardless of the phase of the clock. Only a few genes (13, namely *per2*, *cry1a*, *tef1*, *bhlhe40 [dec1]*, *lonfr1 [2 of 2]*, *si:ch211-195b13.1 [serum/glucocorticoid regulated kinase 1-like]*, *carboxyl ester lipase*, *guca1c*, and *zgc:73369 [CCDC58]*) showed upregulation after 1 h of light. However, after 3 h, we found 74 genes (80 probes) to be up- and 24 genes to be downregulated in the larvae (Figure 2). Most upregulated genes of the entire set are encountered in the larvae subset, most likely reflecting the higher complexity of the organism and a potential contribution of systemic factors. Also the vast majority of downregulated genes from the entire set is found only in the larvae, including *ndrg1l*, the most strongly downregulated gene in the entire set.

To examine genes regulated at the organ level, we made use of the ease with which adult zebrafish hearts can be kept in tissue culture. The dissected hearts were kept for 4 days in constant darkness to desynchronize their clocks and then subjected to a 1- or 3-hour light pulse. Again, we found very few genes (*ddb2* and *zorba*) regulated after 1 h, and more (12 [14 probes]) regulated after 3 h (Figure 2). Overlap with the larval set was high, with just two genes (*zorba* and *clock3*) regulated only in the heart culture, but not in the larvae. Adult hearts are less transparent than the larvae, thus the lower number of induced genes might also reflect a lower intensity of light that reaches the cells within the tissue, attenuating the response.

To examine which genes would be regulated cell autonomously by the light pulses, we also examined the effect of light exposure on cells of a zebrafish tissue culture cell line (PAC2 [16]). Cells were kept in constant darkness for a week prior to the light pulses in order to desynchronize their clocks. We found two genes (*lonfr1 [2 of 2]* and the novel protein *si:ch211-284a13.1*) induced after 1 h of light, while 30 genes (34 probes) were regulated after 3 h (29 [33 probes] upregulated and 1 downregulated, Figure 2). Overall, the number of regulated genes is lower than in the larvae, but higher than in the hearts. This may reflect the lower complexity and higher homogeneity in the cell culture when compared with the organ and whole organism levels. Only relatively few genes (11 [12 probes]) are specific to the cell culture set, including *si:ch211-284a13.1*.

**Figure 3:**
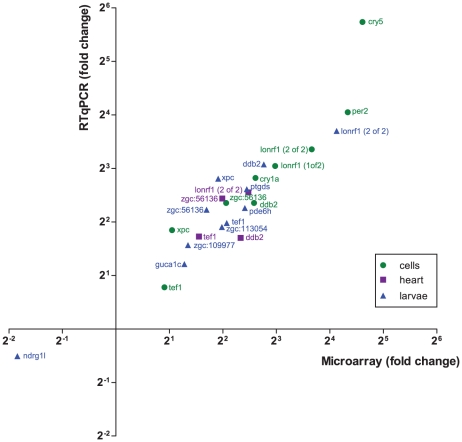
qPCR validation of the microarray results. Graph indicating the correlation of light induced fold change values as determined by RTqPCR (y-axis) and microarray (x-axis) for 24 gene-treatment combinations. Values are given for genes tested in cells (green circles), hearts (purple squares) and larvae (blue triangles). The correlation is statistically highly significant (Spearman *r* = 0.9009, *p*<0.0001).

In total, 124 probes corresponding to 117 genes were found to be regulated, with 27 genes downregulated and 90 (97 probes) upregulated. 8 genes fit to several probes in the set, namely *cry-DASH*, *zgc:56136*, *tef1*, *per2*, *xpc*, *pde6h*, *ptgds* and *zgc:153154*. We selected a subset of genes for validation by qPCR. As shown in Figure 3, the qPCR results correlate well with the chip results (Spearman r = 0.9009, *p*<0.0001), indicating a high level of confidence for the identified gene set.

### Cluster analysis

Hierarchical clustering of the differentially expressed probe set reveals that most probes fall into one of five categories with similar regulation (Figure 2): Probes that are upregulated globally (in cells, hearts and larvae), those upregulated only in larvae and cells, those upregulated in larvae only or in cells only, and those downregulated in larvae only. Overall, fewer genes are regulated after just 1 h of light, thus the clusters are mainly determined by the values attained after 3 h of light. Only very few genes (1-2 each) belong to other patterns of expression (e.g. up in hearts and cells, in hearts only, after 1 h only, first up and then down, down in cells only, down after 1 h only). The largest group of upregulated genes (53) constitutes the “larvae only” group, while the “larvae down” group contains the vast majority of downregulated genes (24). (The only downregulated gene not found in the larvae is *dbp1*, which is only found in the cell set.) The “global”, “larvae and cells” and “cells only” groups each contain a similar number of genes (10, 13 and 10, respectively).

### Functional classes of light regulated genes

**Figure 4:**
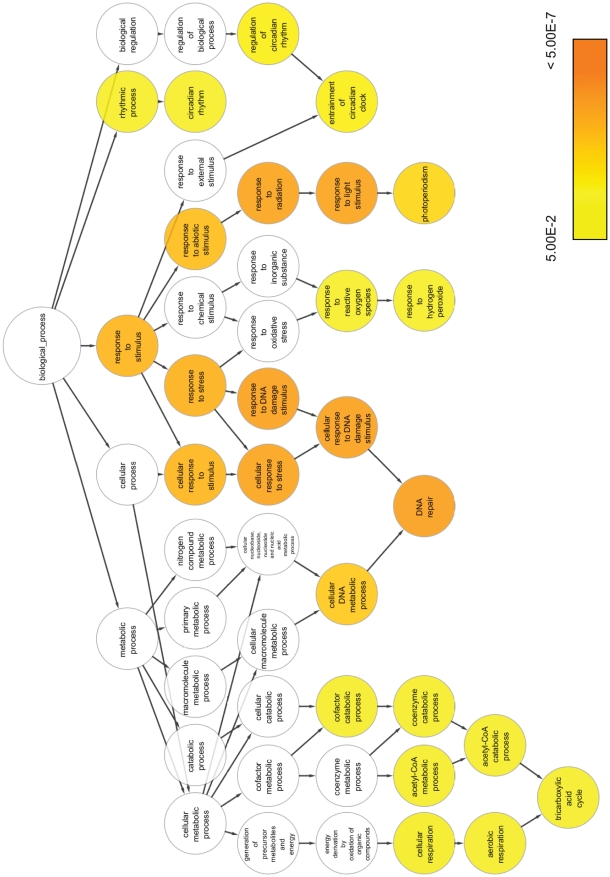
Gene Ontology hierarchy and enrichment statistics for the biological process ontology. GO terms within the biological process ontology that are significantly enriched (adjusted *p*≤0.05) in the light induced gene set are indicated in colour, with the colour shade corresponding to the enrichment *p*-value.

In order to evaluate which types of functions would be most prominent in the light regulated gene sets, we performed an analysis for enrichment of specific Gene Ontology (GO) categories within the ontologies of “biological process”, “molecular function” and “cellular component”. The upregulated and the downregulated gene sets were compared with all genes that were present on the Affymetrix array and were expressed in at least one of the examined conditions. This controlled for any bias in GO categories arising from the selection of genes on the chip and the choice of tissues or cell types examined. Although this analysis is limited by the currently still incomplete annotation of zebrafish genes, it allowed us to identify several statistically enriched functions in our light induced gene set (Table S3). The hierarchical organization of the three gene ontology fields is shown in Figure 4 (biological process), Figure S1 (molecular function) and Figure S2 (cellular component), together with the significance of enrichment in the light induced gene set indicated by colour. In contrast to the upregulated genes, the downregulated genes appear more heterogenous with respect to their functions, and indeed no GO category was found to be enriched in this set (data not shown). With the caveat that the functions of zebrafish and human orthologues may have diverged considerably during evolution, we also made an attempt to exploit the better gene annotation available for human genes. We identified human orthologues of our genes (Table S4) and annotated this list with data mining tools. We mined the set for enriched MeSH disease terms (with LitInspector) and for KEGG pathways (using ClueGO), thereby linking these orthologues with human diseases or metabolic and signalling pathways, respectively. Terms from the two databases that were statistically enriched in our set are shown in Table S7 and Table S8, together with the corresponding genes.

### Circadian clock genes

As expected, we encountered a number of circadian clock genes among the upregulated genes at all levels of organisation. The GO analysis reveals that, in the biological process field, categories related to “circadian rhythm”, such as “regulation of circadian rhythm” and “photoperiodism”, are clearly overrepresented (Figure 4 and Table S3), and also the KEGG search with human homologues yields “circadian rhythm” as an enriched term (Table S7). Indeed, the most highly upregulated gene of the entire set is *per2* (induced 30 fold in larvae after 3 h and 23 fold in cells). Also two other core clock genes are upregulated in both cells and larvae: *cry1a* and *cry2a*. These genes do not appear to be regulated in the heart 3 h set, potentially indicating differential regulation or robustness of light responsiveness for different clock genes in different tissues. Effectively, three other core clock genes appear as regulated in larvae (*bmal2*), hearts (*clock3*) and cells (*cry3*) only. This differential regulation might include kinetic aspects: *per2* and *cry1a* are upregulated already after 1 h in larvae, but not in cells, and the accessory clock loop gene *dec1/bhlh40* appears to be only transiently upregulated after 1 h in the larvae. Differences in the response of clock genes to entrainment signals in different tissues have also been observed in mammals, where e.g. *per2* responds differently to glucocorticoid changes in certain brain regions (reviewed in [2]). Six of the eight regulated clock genes belong to the negative limb of the feedback loop, suggesting that the effects of light on the clock are predominantly mediated via this part of the oscillator mechanism. In support of this prediction, the GO term “transcription repressor activity” from the molecular function field is enriched (Figure S1 and Table S3), again suggesting that the light input to clock entrainment may act via transcriptional repressor functions.

### DNA repair genes

Another striking set of genes, which includes many of those that fall into the globally upregulated cluster, has functions linked with DNA repair. Enriched GO terms in this functional class come both from “biological process” (“response to DNA damage stimulus”, “DNA repair”, “cellular DNA metabolic process”) and “molecular function” (“DNA photolyase activity”, “damaged DNA binding”) (Figure 4, Figure S1, Table S3). Consistently, *cry5*, also called *6,4 photolyase*, has previously been described to be light induced in the early zebrafish embryo [28] and in zebrafish cell culture [29]. The function of this enzyme is to repair so-called 6–4 photoproducts created by UV light exposure. Our microarray results extend this observation to the larvae and adult hearts as well as to another cell culture line, the PAC2 cell line. Also *cry-dash* has been implicated in DNA repair, since *cry-dash* homologues from the bacterium *Vibrio cholera*, the plant *Arabidopsis thaliana* and the frog *Xenopus laevis* have been found to function as photolyases specific for cyclobutan pyrimidine dimers in single stranded DNA [51]. Another example is *ddb2*, which binds to damaged DNA that has been distorted by bulky lesions or cyclic pyrimidine dimers generated by exposure to UV light. DDB2 forms part of a complex with the Xeroderma pigmentosum C (XPC) protein which triggers nucleotide excision repair (reviewed in [52], [53]). Strikingly, *xpc* itself is also induced by light in cells and larvae. *ddb2* is already weakly induced in cultured hearts after 1 h of light exposure. Furthermore, *xrcc1* (*X-ray repair cross complementing protein 1)*, which is induced only in the larvae, is involved both in base excision repair (reviewed in [54]) and in nucleotide excision repair, suggesting that a whole set of genes contributing to this pathway is coordinately regulated by light. This is also reflected by the fact that the category “nucleotide excision repair” appears as enriched in the KEGG pathway analysis (Table S7).

Another gene induced in the larvae is *dclre1a* (*DNA cross link repair 1A)*, which is involved in the repair of interstrand cross-links. Also the novel protein encoded by *zgc:66475* might be related to DNA repair, as it contains a DNA photolyase class 2 domain, and the *coiled-coil domain-containing protein 58* (*ccdc58*) gene contains a *caffeine induced death protein 2* domain, which apparently is involved in caffeine mediated disruption of the DNA replication checkpoint of the cell cycle. Finally, the *LOC570063* gene shows homology to *Xenopus wdr76*, the human homologue of which has been shown to interact with the ubiquitin-ligase Cul4-DDB1, a regulator of DNA damage response, replication and cell cycle progression [55]. Thus, it appears that a comprehensive set of genes involved in DNA repair is upregulated by visible light exposure in the zebrafish. This suggests that DNA damage response pathways are not only triggered by UV exposure, but that these genes already respond to visible wavelengths of light, perhaps in an adaptive response that reflects anticipation of UV exposure under natural lighting conditions. These findings are consistent with an earlier report that embryos exposed to light during early development are more resistant to DNA damaging irradiation as the result of light driven induction of 6,4 photolyase [28]. Our results suggest that indeed a whole battery of DNA damage repair related genes forms part of this light response.

### Stress response genes

The DNA repair pathways form part of the cell stress response, and also other stress related proteins are present in the set, as further reflected by the enrichment of categories within the field “response to stress” in the GO analysis (Figure 4 and Table S3). Thus, both the heat shock chaperones *hsp90a.2* and *hspd1* and their transcriptional regulator *heat shock factor 2*, *hsf2*, are induced in the larval set. This might indicate at least a partial overlap between the responses to light signals with those to heat signals. Heat shock proteins and the Heat shock factor 1 have been implicated in entrainment of the circadian clock by temperature [56], [57], and our data thus raise the possibility that these two entrainment signals converge on similar pathways. Heat shock proteins can also be induced by oxidative stress [58], and interestingly, several genes involved in the response to oxidative stress are also upregulated. Thus, *zgc:110343* (*peroxiredoxin-1*) and *gclc* (*glutamate-cysteine ligase, catalytic subunit*) appear as upregulated transcripts in the cells only. *gclc* catalyzes a step in glutathione biosynthesis, which is a major cellular antioxidant, and *peroxiredoxin-1* reduces peroxides. In the larvae, *zgc:56005* (*oxidative stress induced growth inhibitor 1*) is upregulated, which has been implicated in the regulation of apoptosis. Interestingly, reactive oxygen species (ROS) generated by flavin containing proteins upon light exposure have been suggested as one potential signal mediating light induced gene expression in the zebrafish [59]. It is tempting to speculate that the induction of genes involved in buffering ROS might prepare the organism for the elevated oxidative stress levels generated by sunlight, but could at the same time function in desensitizing the light signalling pathway itself via a negative feedback mechanism that degrades the ROS signals.

### Other enriched functional classes

#### Heme metabolism

Quite a number of genes appear to be involved in various metabolic pathways. A relatively large subset of these genes functions in the mitochondria. For example, the *ferrochelatase* (*fech*) catalyses the last step in heme biosynthesis on the inner mitochondrial membrane, and also the *mitochondrial translocator protein* (*mtso*) is involved in heme and steroid biosynthesis. The KEGG pathway analysis confirms an enrichment of genes linked with the category “Porphyrin and chlorophyll metabolism” (Table S7). Heme is also a ligand for the circadian clock proteins Rev-erbα and β, which in turn regulate transcription of the rate limiting enzyme of heme synthesis [60]. Thus, the possibility emerges that light might also affect the clock via changes in heme metabolism.

#### Mitochondrial genes

Several other mitochondrial genes that are upregulated are involved in the electron transport chain (*cox15 cox17*, *zgc:163000*/*succinate dehydrogenase complex assembly factor 2, sdha, sdhb*) or have been implicated in apoptosis (*LOC557507*/*apoptosis-inducing factor (AIF)-like mitochondrion-associated inducer of death* and *zgc:112986/presenilins-associated rhomboid-like protein*). The enrichment of the GO categories “mitochondrial envelope” and “mitochondrial part” from the cellular component field (Figure S2 and Table S3) and of “mitochondrial diseases” in the MeSH analysis (Table S8) lends further support to a link between the light response and mitochondrial function. This might reflect the multiple roles mitochondria play in ROS metabolism and in apoptosis pathways, which both are involved in phototoxicity processes [61], [62], [63].

#### Retinoid binding genes

The Retinol binding proteins *rbp4* and *rbp4l* and the Prostaglandin D2 synthase *ptgds* [64] *(*as well as the very similar *zgc:153154*) constitute an enriched class of “retinoid binding” in the molecular function GO field (Figure S1 and Table S3). Since retinaldehydes are the chromophores of opsin photoreceptors, it is tempting to speculate that these genes could be involved in opsin turnover. Notably, *rbp4l* is strongly expressed in the zebrafish retina (see also below). Interestingly, the Ptgds enzyme, by way of its product prostaglandin D2, has been implicated in sleep regulation in mammals, thus linking it with an important circadian clock regulated process [65].

### Transcription factors

Many signalling pathways converge upon transcription factors, which integrate signalling information for the transcriptional regulation of downstream target genes. Several transcription factor genes are represented in the set in addition to those which constitute elements of the circadian clock feedback loop, including *pknox2* and *sall1a* (upregulated) as well as *sox9b* (downregulated). The most prominent among these is the *thyroid embryonic factor 1 (tef1*), which is globally upregulated after 3 h and is already induced after 1 h in the larvae. The *tef1* gene, a D-box enhancer element binding factor, has previously been reported to be light induced in zebrafish cells and larvae [25]. It was also implicated in the light induction of *per2* in the larval zebrafish pineal gland via an E- and D-box-containing light responsive element in the *per2* promoter. Our observation that *tef1* is also induced by light in adult heart cultures may suggest a more global role for this factor in mediating light induction (see also below). In addition, another D-box binding factor, *dbp1* [66], is present in the set; however, this factor appears to be downregulated in the cell samples only.

### Genes with unknown functions

Many genes in the set have unknown or still poorly defined functions. Perhaps the most interesting of these genes is one of the two paralogues of the *LON peptidase N-terminal domain and ring finger 1* gene (provisionally named *lonrf1 [2 of 2]*). *lonrf1 (2 of 2)* is upregulated already after 1 h in both cells and larvae and strongly increased after 3 h in all three sets. Strikingly, this is the only gene showing such a strong and robust light induction in the entire set (thus of 15,617 probes on the microarray). Its close relative *lonrf1 (1 of 2)* also shows upregulation in all three sets, but only after three hours. The *lonrf1s* contain predicted TPR repeat domains, which have been implicated in protein-protein interactions. They also contain RING finger domains similar to those found in the *rad18* gene, an E3 ubiquitin-protein ligase involved in postreplication repair of UV-damaged DNA [67]. Furthermore, *lonrf1s* harbour Lon protease domains that have been previously found in bacterial proteases, homologues of which are also found in yeast and human mitochondria [68]. E. coli *lon* has been implicated in various functions, such as radiation resistance and proteolysis of regulatory proteins. Thus, similarly to *rad18*, *lonrf1s* might potentially be involved in DNA repair, or alternatively they might function as a ubiquitin ligase or protease. Interestingly, ubiquitin ligases (and posttranslational regulation in general) have increasingly been recognized as regulators of core components of the circadian clock [69], [70]. Given the close link between light sensing and the circadian clock, it is tempting to speculate that they are also involved in the light input pathway.

### Genes expressed in photoreceptive structures

One set of signalling pathway components upregulated in the larval set appears to be involved in the light reception cascade of the dedicated photoreceptors in retina and/or pineal gland, which are both photoreceptive structures in lower vertebrates: a *cone specific phosphodiesterase 6H* (*pde6h*), a *retinal arrestin 3-like* gene (*arr3l*) and the *guanylate cyclase activator 1C* (*guca1c*). According to ZFIN annotation, also many other genes in our light regulated set are reported to be expressed specifically or at higher levels in the retina and/or the pineal gland. These include the upregulated genes *tef1*, *per2*, *bhlhe40* (*dec1*), *zgc:109977* (*cabp5)*, *rbp4l*, *sdhb*, *hsp90a2*, *hspd1*, *hsf2*, *pknox2*, *zgc:73369* (*ccdc58*), *zgc:113054*, *zgc:85644*, and *scl25a16* as well as the downregulated ones *sox9b*, *syt5a*, *fbxo25*, *cyp3a65*, *ppdpfa*, *ndrg1l*, *ndrg4* and *si:busm1-57f23.1*. In addition, *cox17*, *hsp90a2* and *sepw1* are expressed in the lens of the eye. In further support of a role in eye function for many genes, the term “eye diseases” is also found enriched in the MeSH database analysis. The dedicated photoreceptive structures have to strike a balance between their photoreception function, which requires efficient exposure of their cells to light, and the protection of these cells from the damaging side effects of this light exposure [71]. Interestingly, several of the light regulated genes expressed in the eye appear to be linked to either of these processes. Regulation of their expression might exploit the pathways of peripheral light reception, but could also involve more specific mechanisms linked with dedicated photoreceptor function.

In summary, various functional groups are present in the light regulated gene set. Prominent are genes involved in the circadian clock and DNA repair, but also other stress related genes and genes related to mitochondrial function are to be frequently represented. A substantial number of the genes is reported to be expressed in the eye or in the pineal gland, suggesting a potential link with the dedicated light reception function in these structures.

### Light responsive promoter elements

**Figure 5:**
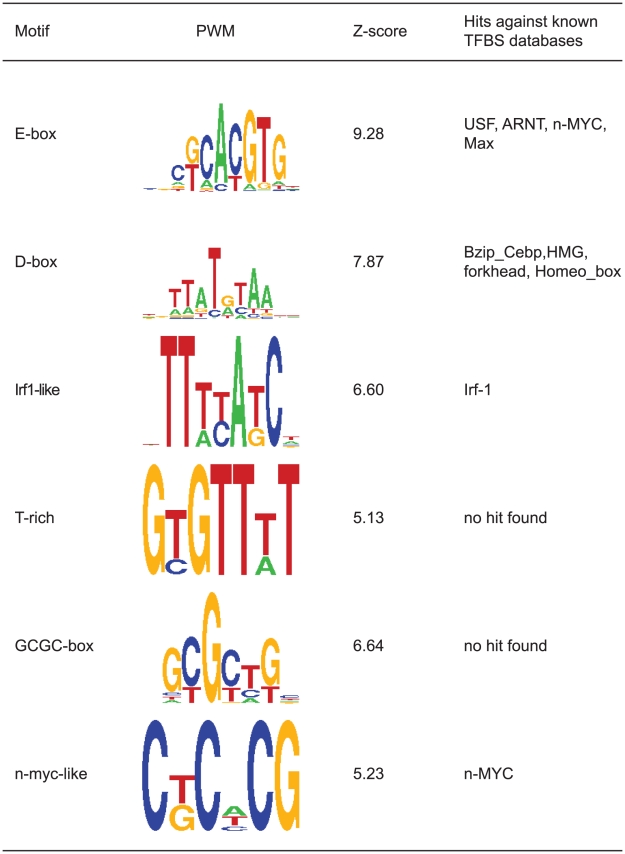
Enriched sequence motifs as identified by Trawler in the promoters of the light induced gene set. Table indicating positional weight matrices (PWM), Z scores and hits of the motifs in databases of known transcription factor binding sites (TFBS).

Do the light upregulated genes identified in this study share a common transcriptional control mechanism? In order to address this fundamental question we chose to examine the promoters of the light regulated genes for enriched transcription factor binding sites and thereby obtain information on the repertoire of light regulated transcriptional control mechanisms. Thus, we examined our upregulated gene set using the Trawler analysis tool suite to determine which short sequence motifs were enriched within 500 bp upstream and 100 bp downstream of the transcription start site. This promoter region was chosen since the light responsive element of the *per2* gene was found within this region [25], and also the AP1 elements suggested to mediate light responsiveness of the *cry1a* and *wee1* genes are located within these boundaries [24]. The retrieved motifs were then examined for matches to known transcription factor binding sites. We identified six motifs that could be found repeatedly when changing parameters of the Trawler search (Figure 5). Strikingly, the most robustly overrepresented motifs in our set match E- and D-boxes, the elements that have previously been implicated in the light response of the *period2* gene [25]. Interestingly, in 44% of the genes where we could detect Trawler defined E- or D-box matrices, both E- and D-boxes were present (Figure S3). Some of these E- and D-box motifs are closely spaced in a manner resembling their location within the *per2* promoter. No AP1 binding sites were among the enriched promoter motifs, indicating that if they do participate in the light regulation of certain genes, they do not seem to play a global role in light regulated transcription.

### Minimal E- or D-box enhancer reporter constructs respond to light in both cells and larvae

**Figure 6:**
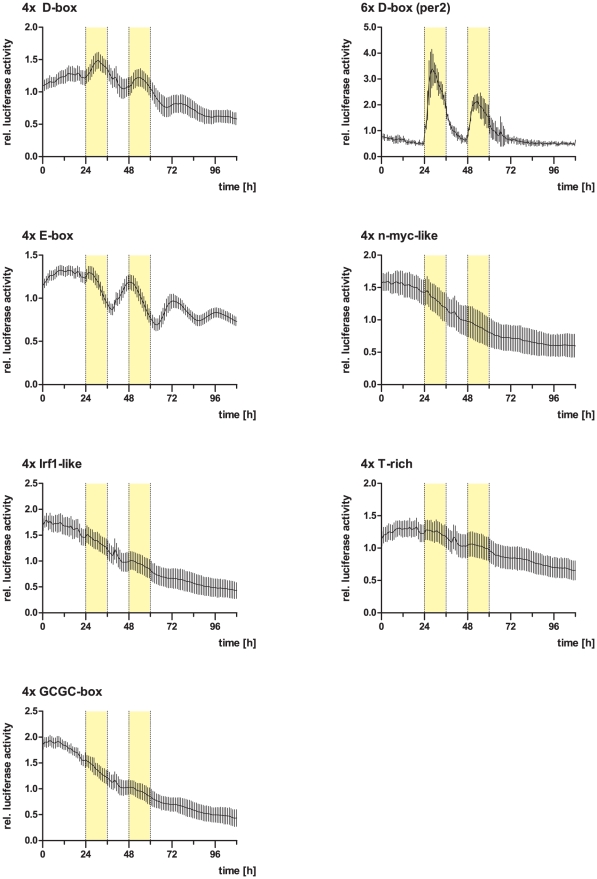
Bioluminescence traces of Pac2 cells containing reporter constructs with concatemerised Trawler motifs. Graphs show relative luciferase activity for the indicated motifs over several days under the indicated light dark regimes. Yellow bars represent times with lights on. Values are means of 8 independent wells, error bars indicate standard deviation. For details see text.

Next, we asked whether the enriched motifs would be able to mediate light induced transcriptional changes in the context of a heterologous promoter construct. For each motif, we concatemerized four consensus sequences separated by a spacer of 10 bp and cloned them into a luciferase reporter vector (Table S6). We transfected PAC2 cells with these constructs, incubated the transfected cells in constant darkness (DD) for three days to desynchronize their clocks and then tested them for light induced luciferase expression by exposing them to a light dark (LD) cycle for two days, followed by a period of two days in DD to identify potential circadian clock regulation. Four motifs (“n-myc-like”, “irf1-like”, “T-rich” and “GCGC-box”) failed to show either light or clock regulation, while both E- and D-boxes were able to mediate a light response (Figure 6). Consistent with previous reports [17], [25], [72], upon exposure to light the 4x E-box construct shows a brief initial rise in luciferase activity, followed by a decrease that persists until the middle of the following dark phase, when it subsequently starts to rise again. This behavior likely reflects the core clock control of gene expression mediated via the E-boxes and the synchronization of the desynchronized oscillators to a common phase by the light pulse [18]. This clock control of E-box expression is also clearly visible by the continuing oscillations after release into DD. The 4x D-box construct shows robust and persistent light induction, which only starts to decrease in the second part of the light phase and thus behaves in a similar fashion to a D-box construct derived from the *per2* promoter (see below, 6x D-box [25]). The expression of the 4x D-box constructs also reveals signs of clock mediated regulation, since oscillations continue after release into DD. In addition, the increase in expression of the 4x D-box construct even before the onset of the subsequent light period is also indicative of a clock contribution to their regulation. Interestingly, the 6x D-box construct based on the *per2* D-box fails to show rhythmic expression following transfer from LD to DD. This suggests that the precise promoter environment may modulate the degree of light and clock regulation of the D-box. The natural promoter environment might also be critical for the function of the remaining enriched promoter elements that did not show light regulation when tested as concatemers within an artificial heterologous promoter. They may interact with the E- or D-boxes or with each other and thus fulfill permissive or enhancing roles that would not be apparent in a minimal promoter-based assay. More detailed promoter analyses will be required to explore how these motifs contribute to light regulated transcription.

**Figure 7:**
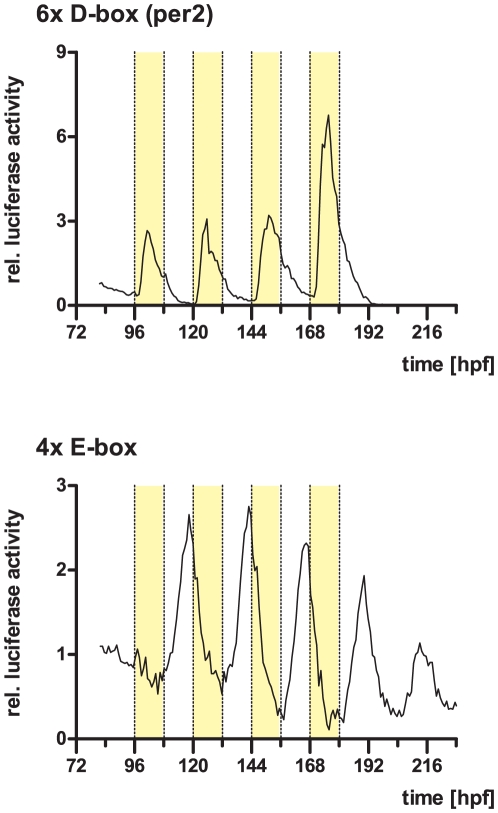
Bioluminescence traces of single larvae injected with E- and D-box reporter constructs. Graphs show relative luciferase activity of a representative single zebrafish larva injected with the indicated reporter construct. Yellow bars represent times with lights on. hpf, hours post fertilization. See text for details.

In order to further examine the general relevance of E- and D-box enhancer elements for light driven gene expression, we tested whether E- and D-boxes would mediate a light response in the context of transgenic zebrafish larvae. Zebrafish embryos were injected with the E- and D-box heterologous promoter reporter constructs and bioluminescence was monitored in single larvae between days 4-10 post-fertilization. Both 4x E-box and 6x D-box reporter transient transgenics exhibited a light regulated bioluminescence pattern. The 6x D-box construct shows a strong light induction followed by a decrease in the second part of the light phase (Figure 7). After release into DD, no oscillatory expression is present, comparable with the expression profile of this construct in cultured zebrafish cells. Also the 4x E-box construct reveals a similar expression profile as seen in cell culture, with decreasing expression during the light phase, rising expression in the dark phase which peaks shortly before the end of the phase, and continuing oscillations after subsequent release into DD. Thus, the light and clock regulation mediated by these motifs is also evident in the context of the whole animal.

### Two tef homologues mediate light induction in a differential and gene-specific manner

**Figure 8:**
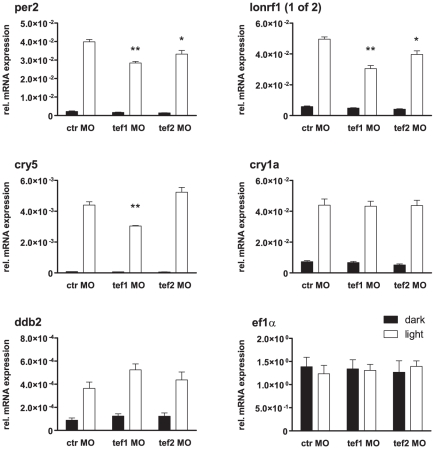
Morpholino mediated knock down of *tef1* and *tef2* differentially affects the light response in PAC2 cells. Bar diagrams showing the expression levels as measured by RTqPCR of the indicated genes under dark control (black) and after a 3-hour light pulse (white) for control morpholino (ctr MO), *tef1* morpholino (tef1 MO) and *tef2* morpholino (tef2 MO) treated cells. Light induced expression of *per2* and *lonrf (1of2)* is attenuated by both *tef1* and *tef2* knockdown, *cry5* is affected only by *tef1* knockdown, and *ddb2* and *cry1a* are affected by neither. *ef1a* serves as control for a non-light responsive gene. Values represent fold *β-actin* mRNA levels. Statistical significance of expression differences to the light control was determined by an unpaired t test and is indicated as follows: **, *p*≤0.01; *, *p*≤0.05.

The zebrafish genome contains two *tef* genes, *tef1* and *tef2* [66]. While *tef1* mRNA expression is induced by light ([25] and this study), *tef2* mRNA expression was found to peak rather at the end of the night under LD cycles [66]. Tef1 has previously been shown to mediate light regulated *per2* expression in the larval pineal gland [25]. To test whether either of the two genes contributes more generally to the cell autonomous light induction of gene expression in zebrafish, we performed morpholino mediated knock-down studies in the zebrafish cell culture system. Cells were exposed to two days of constant darkness to desynchronize their clocks and then received a 3-hour light pulse before harvesting and RNA preparation. Light induced endogenous gene expression was tested by real time qPCR. We examined expression of *per2*, *cry5*, *ddb2*, *cry1a*, *lonrf1 (1of2)* and *ef1a* (Figure 8). Knockdown of the two *tefs* showed differential and gene-specific effects on light induced expression, with *per2* and *lonrf1 (1of2)* expression attenuated by both *tef1* and *tef2* knockdown, *cry5* affected only by *tef1* knockdown, and *ddb2* and *cry1a* not affected by knockdown of either *tef1* or *tef2*. In general, the *tef1* knockdown effect appeared more pronounced than the effect of the *tef2* knockdown. Thus, *tef1* appears to be not the only mediator of light induced cell autonomous gene expression in PAC2 cells. It may act redundantly with other D-box binding factors, such as *tef2*, or with other as yet unidentified light signalling pathways. Together with the overlapping expression patterns of various D-box binding factors in the zebrafish embryo [66], this also suggests that the two Tef factors may act differentially in various tissues. Very recently, *tef1* knock-down has been reported to globally impair light induced gene expression compared with uninjected controls in 9 hours post fertilization zebrafish embryos that were raised in constant light [73]. This might reflect dependency of light induced gene expression on *tef1* either in certain tissues, which could dominate the light induction in whole embryo RNA samples, or generally after prolonged light exposure. However, in the absence of a comparison to control morpholino injections, it cannot be excluded that the morpholino injection itself might have had unspecific effects which perturbed also the light response.

In summary, our work reveals the light regulated transcriptome of a vertebrate at three levels of organization. Light regulated genes are more likely to function in circadian clock regulation, DNA repair, stress response and mitochondrial processes than other genes. The coordinated light induction of DNA repair genes, particularly of the nucleotide excision repair pathway, suggests that exposure to visible light prepares the animal for the repair of UV induced damage during the day time. Also the response to heat and oxidative stress appears to involve such light induced preparative gene expression. Furthermore, two previously undescribed *lon* peptidase related genes, the two *lonrf1* paralogues, are pervasively upregulated and might thus play an important role in the light response. The enrichment of both E- and D-box elements in the promoters of the light induced genes, which appear together in about half of the promoters, suggests that the factors involved in light mediated expression of *period2* may be part of a more widespread mechanism of light induced gene expression. The enrichment of E-boxes further indicates that the circadian clock and the light response are closely intertwined and cooperate in the regulation of many light induced genes. Knock-down studies of D-box binding Tef factors reveal a gene-specific involvement of *tef1* and *tef2* in the cell autonomous mediation of the light response. Thus, it appears that the light regulated gene expression is mediated by a complex combination of factors that includes the various D-box binding proteins and the core clock, but might also involve signalling through the other promoter elements that are enriched in the promoters of our gene set. This multiple, combinatorial control might be required to mediate gene- and tissue specific light responses while at the same time ensuring robustness of the response. Further work exploring how the different elements of the light response interact on endogenous promoters should reveal the regulatory code underlying light induced gene expression. Strikingly, the evolutionary conservation of some of the elements ([25] and data not shown) in mammals suggests that regulatory principles involved in light regulated gene expression might be conserved in the absence of peripheral photoreception. It will be interesting to determine which endogenous signals act via such elements in mammals and if they are linked with functions similar to those of the light induced zebrafish genes.

## Supporting Information

Figure S1 **Gene Ontology hierarchy and enrichment statistics for the molecular function ontology**. GO terms within the molecular function hierarchy that are significantly enriched (adjusted *p*≤0.05) in the light induced gene set are indicated in colour, with the colour shade corresponding to the enrichment *p*-value.
(PDF)

Figure S2 **Gene Ontology hierarchy and enrichment statistics for the cellular component ontology**. GO terms within the cellular component hierarchy that are significantly enriched (adjusted *p*≤0.05) in the light induced gene set are indicated in colour, with the colour shade corresponding to the enrichment *p*-value.
(PDF)

Figure S3 **Location of E- and D-box elements in the promoters of the light induced gene set**. The positions of E-box (light blue boxes) and D-box (dark blue boxes) motifs in the promoter regions of the light induced gene set are indicated. Only the instances with a position *p*-value below 0.0001 as defined by MAST are shown. 44% of the promoters contain both elements, frequently closely spaced similar to the arrangement found in the light responsive module of the *per2* promoter.
(PDF)

Table S1 **Summary of expression values and gene annotations from the microarray analysis**. “Gene symbol” denotes the symbol used throughout the text, the figures and the tables. “Gene name (manually)” gives the manually annotated gene name associated with each probe. For each symbol and name it is indicated from which database the label was derived. M values denote the mean value of fold up- or downregulation in log_2_ format. For each fold change value, the statistical significance values are given in columns S to AP.
(XLS)

Table S2 **Summary of chromosomal location and annotation of the promoter sequences from the upregulated gene set used for the Trawler analysis.**
(XLS)

Table S3 **Summary of Gene Ontology analysis.** This table summarizes the Gene Ontology analysis results illustrated in Figure 4, Figure S1 and Figure S2. “*p*-value” denotes the significance values of hypergeometric test for the enrichment of the corresponding GO term, “corrected *p*-value” denotes the value after FDR correction.
(XLS)

Table S4 **List of human orthologues of the light regulated gene set.**
(XLS)

Table S5 **qPCR primer sequences.**
(XLS)

Table S6 **Sequences of oligonucleotides used for cloning of Trawler motif reporter constructs.**
(XLS)

Table S7 **Summary of KEGG pathway analysis results**
(XLS)

Table S8 **Summary of MESH term analysis results**
(XLS)
