# Supplementary figures and images for: The Light Responsive Transcriptome of the Zebrafish: Function and Regulation
Source: PLoS One. 2011 Feb 15;6(2):e17080. doi: 10.1371/journal.pone.0017080 (PMC3039656; doi:10.1371/journal.pone.0017080)

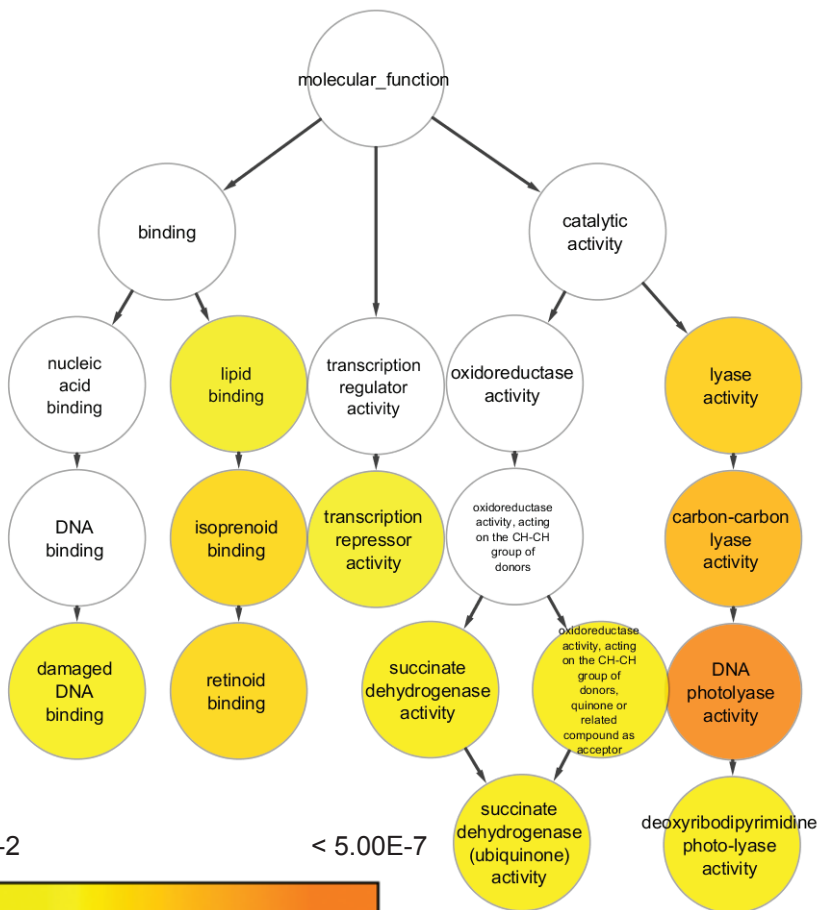

Supplement: Figure S1 — Gene Ontology hierarchy and enrichment statistics for the molecular function ontology. GO terms within the molecular function hierarchy that are significantly enriched (adjusted p≤0.05) in the light induced gene set are indicated in colour, with the colour shade corresponding to the enrichment p-value. (PDF) [file pone.0017080.s001.pdf]

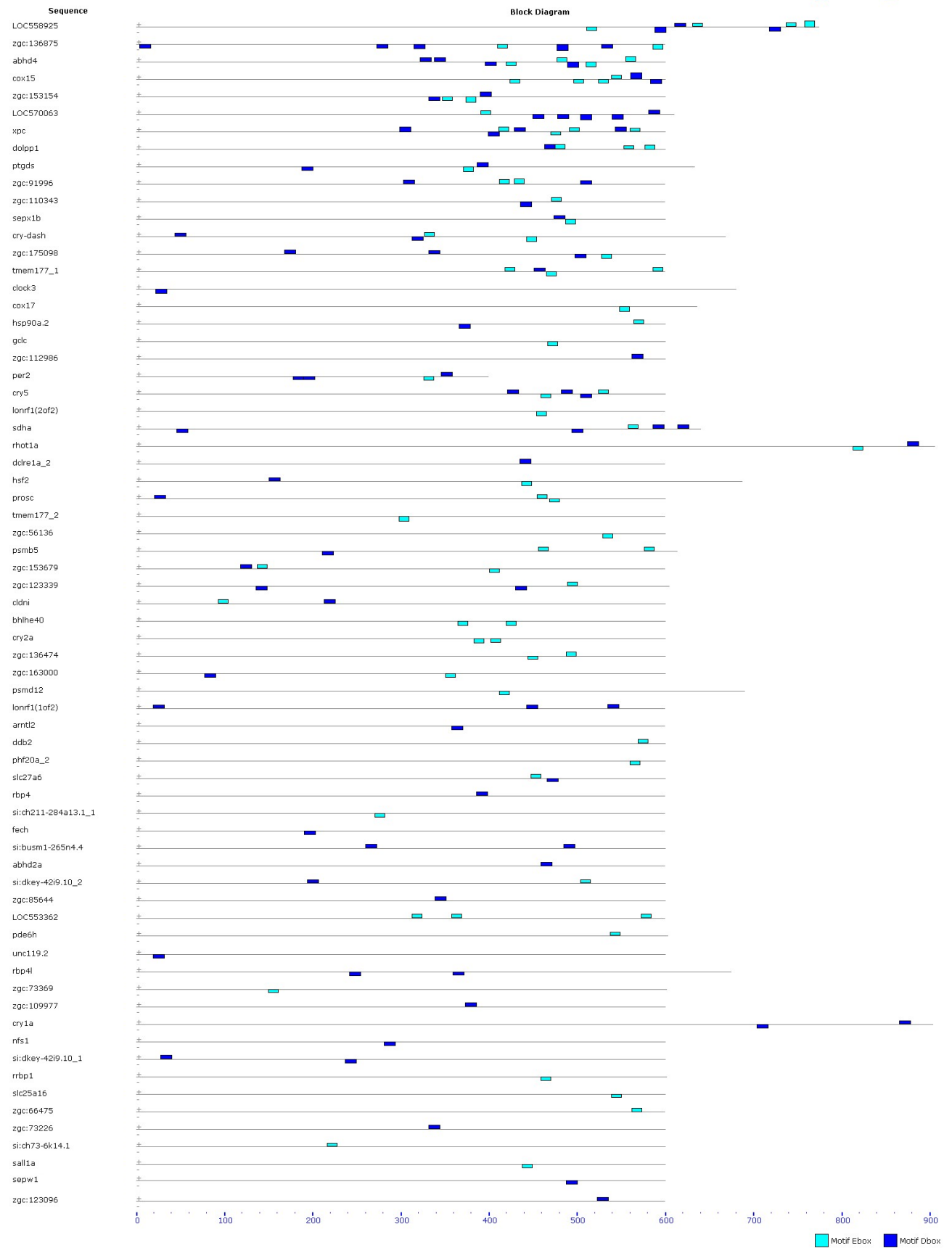

Supplement: Figure S3 — Location of E- and D-box elements in the promoters of the light induced gene set. The positions of E-box (light blue boxes) and D-box (dark blue boxes) motifs in the promoter regions of the light induced gene set are indicated. Only the instances with a position p-value below 0.0001 as defined by MAST are shown. 44% of the promoters contain both elements, frequently closely spaced similar to the arrangement found in the light responsive module of the per2 promoter. (PDF) [file pone.0017080.s003.pdf]
