# Supplementary material for: The Light Responsive Transcriptome of the Zebrafish: Function and Regulation
Source: PLoS One. 2011 Feb 15;6(2):e17080. doi: 10.1371/journal.pone.0017080 (PMC3039656; doi:10.1371/journal.pone.0017080)
Supplement: Figure S2 — Gene Ontology hierarchy and enrichment statistics for the cellular component ontology. GO terms within the cellular component hierarchy that are significantly enriched (adjusted p≤0.05) in the light induced gene set are indicated in colour, with the colour shade corresponding to the enrichment p-value. (PDF) [file pone.0017080.s002.pdf]

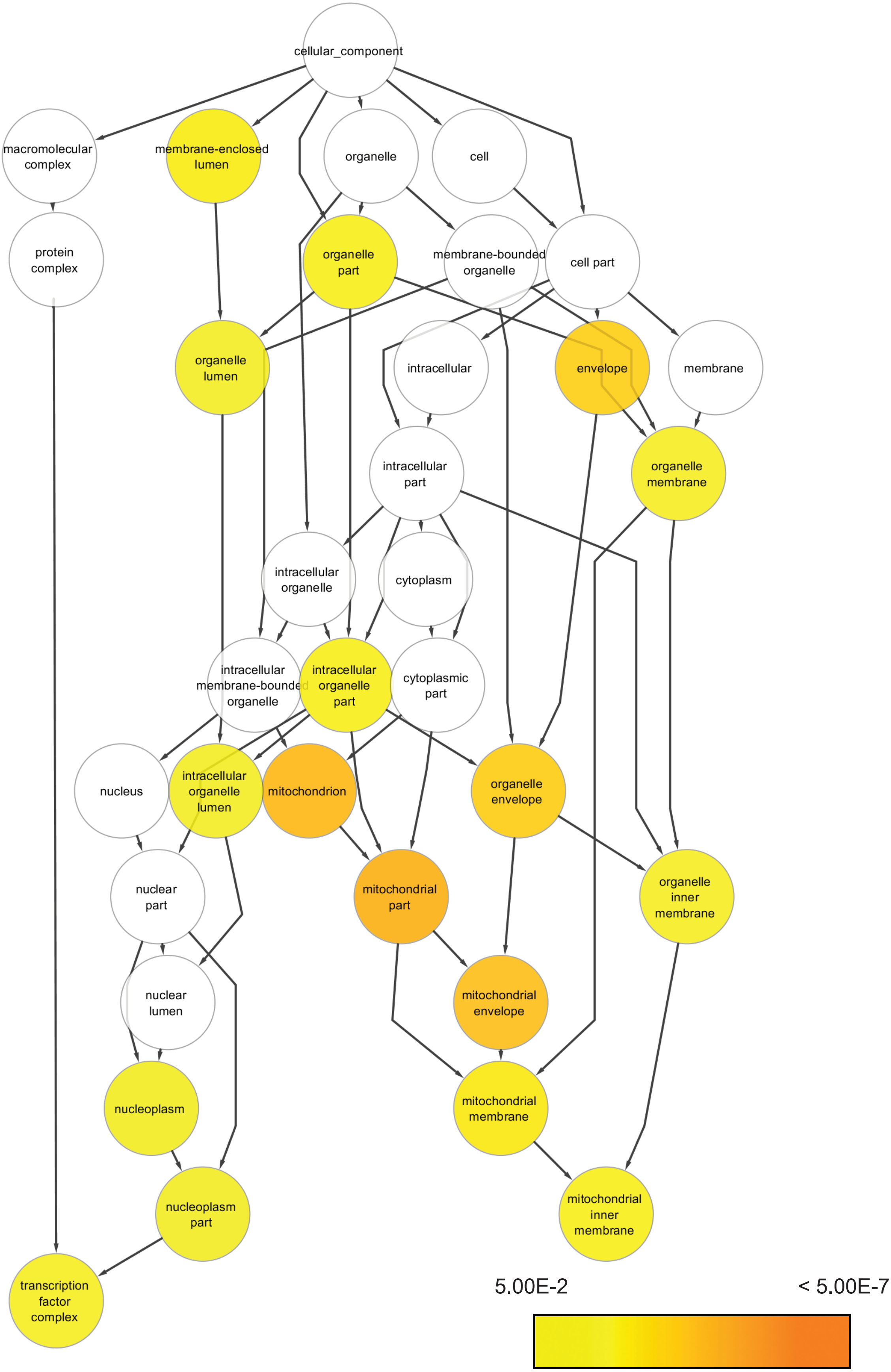
cellular_component
macromolecular complex
membrane-enclosed lumen
organelle
cell
protein complex
organelle part
membrane-bounded organelle
cell part
organelle lumen
intracellular
envelope
membrane
intracellular part
organelle membrane
intracellular organelle
cytoplasm
intracellular membrane-bounded organelle
intracellular organelle part
cytoplasmic part
nucleus
intracellular organelle lumen
mitochondrion
organelle envelope
nuclear part
mitochondrial part
organelle inner membrane
nuclear lumen
mitochondrial envelope
nucleoplasm
mitochondrial membrane
nucleoplasm part
mitochondrial inner membrane
transcription factor complex
5.00E-2
< 5.00E-7
